# Identification of low risk of violent crime in severe mental illness with a clinical prediction tool (Oxford Mental Illness and Violence tool [OxMIV]): a derivation and validation study

**DOI:** 10.1016/S2215-0366(17)30109-8

**Published:** 2017-06

**Authors:** Seena Fazel, Achim Wolf, Henrik Larsson, Paul Lichtenstein, Susan Mallett, Thomas R Fanshawe

**Affiliations:** a Department of Psychiatry, Warneford Hospital, University of Oxford, Oxford, UK; b Department of Medical Epidemiology and Biostatistics, Karolinska Institutet, Stockholm, Sweden; c School of Medical Sciences, Örebro University, Örebro, Sweden; d School of Population and Health Sciences, University of Birmingham, Birmingham, UK; e Nuffield Department of Primary Care Health Sciences, University of Oxford, Oxford, UK

## Abstract

**Background:**

Current approaches to stratify patients with psychiatric disorders into groups on the basis of violence risk are limited by inconsistency, variable accuracy, and unscalability. To address the need for a scalable and valid tool to assess violence risk in patients with schizophrenia spectrum or bipolar disorder, we describe the derivation of a score based on routinely collected factors and present findings from external validation.

**Methods:**

On the basis of a national cohort of 75 158 Swedish individuals aged 15–65 years with a diagnosis of severe mental illness (schizophrenia spectrum or bipolar disorder) with 574 018 patient episodes between Jan 1, 2001, and Dec 31, 2008, we developed predictive models for violent offending (primary outcome) within 1 year of hospital discharge for inpatients or clinical contact with psychiatric services for outpatients (patient episode) through linkage of population-based registers. We developed a derivation model to determine the relative influence of prespecified criminal history and sociodemographic and clinical risk factors, which are mostly routinely collected, and then tested it in an external validation. We measured discrimination and calibration for prediction of violent offending at 1 year using specified risk cutoffs.

**Findings:**

Of the cohort of 75 158 patients with schizophrenia spectrum or bipolar disorder, we assigned 58 771 (78%) to the derivation sample and 16 387 (22%) to the validation sample. In the derivation sample, 830 (1%) individuals committed a violent offence within 12 months of their patient episode. We developed a 16-item model. The strongest predictors of violent offending within 12 months were conviction for previous violent crime (adjusted odds ratio 5·03 [95% CI 4·23–5·98]; p<0·0001), male sex (2·32 [1·91–2·81]; p<0·0001), and age (0·63 per 10 years of age [0·58–0·67]; p<0·0001). In external validation, the model showed good measures of discrimination (c-index 0·89 [0·85–0·93]) and calibration. For risk of violent offending at 1 year, with a 5% cutoff, sensitivity was 62% (95% CI 55–68) and specificity was 94% (93–94). The positive predictive value was 11% and the negative predictive value was more than 99%. We used the model to generate a simple web-based risk calculator (Oxford Mental Illness and Violence tool [OxMIV]).

**Interpretation:**

We have developed a prediction score in a national cohort of patients with schizophrenia spectrum or bipolar disorder, which can be used as an adjunct to decision making in clinical practice by identifying those who are at low risk of violent offending. The low positive predictive value suggests that further clinical assessment in individuals at high risk of violent offending is required to establish who might benefit from additional risk management. Further validation in other countries is needed.

**Funding:**

Wellcome Trust and Swedish Research Council.

## Introduction

Although absolute risks of people with schizophrenia spectrum and bipolar disorder committing violent crime are typically around 5–10% within 5 years of diagnosis and most patients are not violent in their lifetimes,^1^, ^2^ violence perpetrated by individuals with these disorders is an important preventable cause of morbidity. Furthermore, it contributes to stigma and the large numbers of people with mental illness in prisons.

One of the main approaches to reduce violence risk has been to use structured risk assessment tools, which range from checklists to complex decision trees, and to stratify individuals into high-risk and low-risk groups. These tools are used in mental health services, especially in forensic psychiatry, and are recommended in clinical guidelines.^3^, ^4^, ^5^ Such stratification can help target resources, tailor treatment and risk management, and inform decisions about assertive community treatment, hospital treatment, and other services.^5^

Current risk assessment instruments, however, have been limited by low-to-moderate accuracy,^6^ poor reporting standards,^7^ and inconsistent definitions of what constitutes high risk.^8^ The tools have rarely been developed in individuals with psychosis.^9^ Additionally, many have considerable resource implications, with current approaches taking around 16 person-hours in one forensic psychiatric setting,^10^ and most instruments requiring several hours. By contrast, some areas of medicine, in particular cardiovascular medicine, have developed scalable risk prediction scores, such as the Framingham Risk Score and QRISK prediction algorithm, which can be used in primary and secondary care to inform discussions between clinicians and individuals about risk.^11^ A key factor in their widespread use is their ease and simplicity. The need for shorter violence risk assessments than at present, validated in appropriate patient groups, has been highlighted by an American Psychiatric Association taskforce.^5^ To address the need for a scalable and valid tool to assess violence risk in patients with schizophrenia spectrum and bipolar disorder, we describe the derivation of a score based on routinely collected factors and present findings from external validation.

**Research in context**

**Evidence before this study**

We searched MEDLINE from Jan 1, 1946, until Feb 6, 2017, with no language or date restrictions, for systematic reviews comparing violence risk assessment tools in individuals with a diagnosis of severe mental illness (schizophrenia, schizophrenia spectrum disorder, bipolar disorder, and other psychotic illnesses). We used the following search terms: “violen*” AND (“risk” OR “assess*” OR “predict*” OR “tool*” OR “instrument*”) AND (“mental*” OR “psychiatr*”) AND “systematic review”. We identified two systematic reviews of violence risk instruments in psychosis. The first review, published in 2011, was based on ten commonly used tools for violence risk in psychiatric populations and identified only two instruments validated in 861 patients with psychosis. These two instruments reported areas under the curves that ranged from 0·60 to 0·77, with little other information about performance. A second review, published in 2016, was based on violence risk tools in Chinese patients with psychiatric disorders, and the authors found typically poor-to-moderate performance, with areas under the curves of between 0·67 and 0·72 using tools developed in high-income countries. Current thinking in the field accepts that group data can be informative in decision making about individual cases and separates risk assessment from risk reduction or management. A scalable approach to violence risk assessment (Oxford Risk of Recidivism [OxRec] tool) has been published for released prisoners, which includes modifiable risk factors.

**Added value of this study**

We have developed a 16-item tool using mostly routinely collected information, with good measures of calibration and discrimination (c-index of 0·89 [95% CI 0·85–0·93] in external validation), which has been translated into a freely available online calculator. The tool should be used solely by clinicians and has specific questions about previous diagnoses and treatments.

**Implications of all the available evidence**

Violence risk assessment with use of an online tool can serve as an adjunct to clinical decision making in general adult psychiatry, accurately identify patients at low risk of violent offending, and help inform decisions about additional risk management. General adult psychiatric services could use the tool as part of their routine violence risk assessment for new patients rather than local tools, which have no evidence base.

## Methods

### Study design and patients

We did a cohort study of individuals aged 15–65 years with a diagnosis of severe mental illness (here defined as schizophrenia spectrum or bipolar disorder) through linkage of population-based registers in Sweden. We identified a cohort of 75 158 individuals with 574 018 recorded patient episodes (424 842 [74%] outpatient episodes) between Jan 1, 2001, and Dec 31, 2008. The final study cohort consisted of a single inpatient or outpatient visit for each patient, selected at random (with use of the random number generator in Stata version 12), with equal probability. We excluded repeat visits because they complicate model fitting and interpretation. The study was approved by the Regional Ethics Committee at Karolinska Institutet (2009/939-31/5).

### Procedures

We followed up each individual from the day of their patient episode until the first time that they committed a violent offence, death, emigration, or end of follow-up (12 months after their patient episode). We linked individuals to national registers to obtain information about risk factors, with unique personal identification numbers enabling accurate linkage. We obtained sociodemographic factors and information about previous violent crime conviction, psychiatric diagnoses, and dispensed medication and identified parents and siblings of patients to extract historical information (ie, before the current patient episode; appendix pp 1–2).

### Outcomes

The primary outcome was the occurrence of any violent offending within 1 year of hospital discharge for inpatients or clinical contact with psychiatric services for outpatients (patient episode). We did not consider repeat offences by an individual within 1 year. We used conviction data because the Swedish criminal code determines that individuals are convicted as guilty regardless of mental disorder and no plea bargaining is permitted at conviction. We defined violent crime as homicide, assault, robbery, arson, any sexual offence (rape, sexual coercion, child molestation, indecent exposure, or sexual harassment), or illegal threats and harassment.

### Statistical analysis

We derived the model with logistic regression (appendix pp 3–4). On the basis of existing evidence of criminal history and sociodemographic and clinical factors,^12^, ^13^ we grouped variables a priori on the anticipated strength of association with the outcome in decreasing levels of priority (appendix p 5).^14^, ^15^ We excluded covariates with more than 30% missing data. We made an exception for the recent treatment variables, which were unavailable before 2006 only because the Prescribed Drug Register was not available: the missingness mechanism was thus known and thought to be unrelated to the missing values themselves. We imputed missing data via multiple imputation using chained equations.

We assessed the internal validity of the model using bootstrapping to assess its predictive accuracy.^16^ We used bootstrapping to create 100 samples drawn with replacement from the derivation dataset. To test the external validation of the model, we selected at random (using the sample function in R version 2.3.1) a subsample of geographical regions (the validation sample)^17^ on the basis of the residential geographical location of the individual at the time of diagnosis, comprising around one-fifth of the total sample, and removed it from the dataset used to fit the model (the derivation sample). We chose the number of regions for the validation sample to be large enough for a useful assessment of external validity to be made.^18^ The geographical regions and method for selection of the validation sample are described in the appendix (p 6).

In external validation, we summarised predictive accuracy using: first, the concordance index^19^ to assess discrimination (ability of the model to distinguish between those who do and do not commit a violent crime, with a value of 1 meaning perfect discrimination); second, the Brier score^20^ for calibration (model goodness of fit—whether or not the predicted risk is systematically off target, with 0 meaning perfect calibration; the Brier score measures the mean squared difference between the predicted probability and the actual outcome [violent crime or no violent crime]); third, the net reclassification index^21^ (how well a model rightly or wrongly reclassifies patients compared with alternative models); and fourth, sensitivity and specificity based on a 5% threshold of predicted probability. We compared the proportions of predicted and observed events at different levels of predicted probability using a calibration plot. On the basis of research that has found an incidence of violent crime in schizophrenia spectrum disorders at 1 year of 4%,^1^ we prespecified a 5% cutoff for low-to-high risk of violent offending. We used a higher cutoff than this 4% incidence as the previous data were based on a younger age cohort and only on patients with schizophrenia spectrum disorder. We used Stata version 12 and R version 3.2.1 for all analyses. We followed the TRIPOD statement (appendix p 7).

### Role of the funding source

The funders of the study had no role in study design, data collection, data analysis, data interpretation, or writing of the report. The corresponding author had full access to all the data in the study and had final responsibility for the decision to submit for publication.

## Results

**Figure 1:**
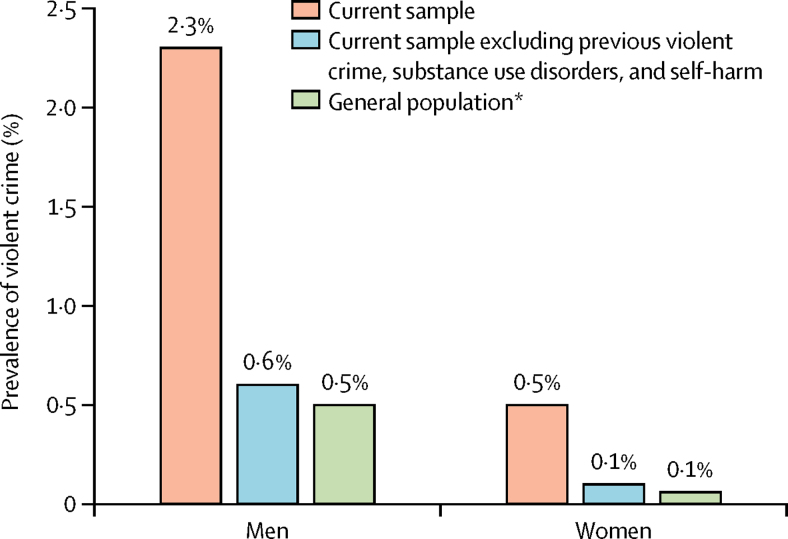
Violent crime over a 12 month period in different populations by sex *Data taken from the general population sample in Fazel and colleagues.^1^

Of the cohort of 75 158 patients with schizophrenia spectrum or bipolar disorder, we assigned 58 771 (78%) to the derivation sample and 16 387 (22%) to the validation sample (table 1). Overall, 869 (2·3%) of 37 221 men and 181 (0·5%) of 37 937 women committed violent crime over a 12 month period (figure 1). In the derivation sample, 830 (1%) individuals committed a violent offence within 12 months of their patient episode, 1702 (3%) died within 12 months, and 40 611 (69%) were outpatients at the time of the episode.

**Table 1:** Baseline characteristics of the derivation sample diagnosed with schizophrenia spectrum or bipolar disorder, with grouping of violence risk factors

		**Patients (n=58 771)**
Group 1*		
Male sex		29 077 (49%)
Age (years)		44 (13)
Previous violent crime		9212 (16%)
Previous drug use		7123 (12%)
Previous alcohol use		8897 (15%)
Previous self-harm		11 510 (20%)
Educational level		
	Lower secondary (<16 years of age)	17 814/50 752 (35%)
	Upper secondary (16–18 years of age)	26 449/50 752 (52%)
	Postsecondary (>18 years of age)	6489/50 752 (13%)
Parental drug or alcohol use		5214/47 957 (11%)
Parental violent crime		3203/47 957 (7%)
Sibling violent crime		4028 (7%)
**Group 2**†		
Diagnosis		
	Schizophrenia spectrum disorder	36 755 (63%)
	Bipolar disorder	22 016 (37%)
Recent treatment (within preceding 6 months)		
	Mood stabiliser	10 390/34 039 (31%)
	Antipsychotic	18 401/34 039 (54%)
	Antidepressant	13 255/34 039 (39%)
	Dependence	1030/34 039 (3%)
Inpatient at time of episode		18 160 (31%)
Length of first inpatient stay >7 days		24 532 (42%)
More than seven patient episodes		16 686 (28%)
**Group 3**‡		
Received benefits§		37 210/57 876 (64%)
Deprivation		
	First decile (lowest)	2793/56 617 (5%)
	Fifth decile	4862/56 617 (9%)
	Tenth decile (highest)	10 769/56 617 (19%)
Never married		34 506/57 459 (60%)
Personal income		
	First decile (lowest)	5444/57 876 (9%)
	Fifth decile	9169/57 876 (16%)
	Tenth decile (highest)	2009/57 876 (3%)
Children in household		11 079 (19%)
Parental psychiatric admission to hospital		13 225/47 957 (28%)
Parental suicide		1417/47 957 (3%)
Comorbid depression		11 934/36 755 (32%)¶
Recent death of family member (within preceding 6 months)		953/47 957 (2%)

Data are n (%), mean (SD), or n/N (%).

* Variables included in the model on the basis of previous evidence.

† Variables considered for the model with strong evidence, but retained in the model if significant.

‡ Variables considered for the model with weaker evidence, but retained in the model if significant.

§ Welfare or disability benefits.

¶ Denominator is the number of patients with schizophrenia spectrum disorder.

We included 16 items in the final model (table 2; appendix p 8). The strongest predictors of violent offending within 12 months were previous violent crime conviction, male sex, and age. The decline in probability of violent offending was approximately linearly related to increasing age (appendix p 9). Personal income and benefit receipt were among the weaker predictors of violent offending. Shrinkage effects (a measure of the adjustment required for a model fitted to sample data so that it does not overestimate predictive performance) were negligible: the average estimate of the shrinkage heuristic from the bootstrapped samples was 99%. We arrived at the final model by including all group 1 variables and the group 2 and 3 variables that retained significance with multivariable analyses.

**Table 2:** Associations between risk factors and violent crime in the derivation sample from the multiple regression model (after multiple imputation)

		**Adjusted odds ratio (95% CI)**	**p value**
Male sex		2·32 (1·91–2·81)	<0·0001
Age at hospital discharge (per 10 years)		0·63 (0·58–0·67)	<0·0001
Previous violent crime*		5·03 (4·23–5·98)	<0·0001
Previous drug use†		1·45 (1·23–1·72)	<0·0001
Previous alcohol use†		1·75 (1·47–2·09)	<0·0001
Previous self-harm†		1·23 (1·04–1·45)	0·02
Educational level		..	0·31
	Lower secondary (<16 years of age)	1 (reference)	..
	Upper secondary (16–18 years of age)	0·88 (0·75–1·04)	..
	Postsecondary (>18 years of age)	0·93 (0·69–1·26)	..
Parental drug or alcohol use†		1·11 (0·91–1·35)	0·30
Parental violent crime*		1·16 (0·92–1·46)	0·21
Sibling violent crime*		0·90 (0·71–1·13)	0·35
	Recent treatment‡—antipsychotic	0·62 (0·51–0·77)	<0·0001
	Recent treatment‡—antidepressant	0·80 (0·65–0·99)	0·04
	Recent treatment‡—dependence§	1·78 (1·22–2·60)	0·003
Inpatient at time of episode		1·37 (1·18–1·59)	<0·0001
Received benefits¶		1·42 (1·17–1·72)	0·0003
Personal income		..	0·046
	Fifth decile	0·84 (0·65–1·10)	..
	Tenth decile (highest)	0·88 (0·50–1·57)	..

* Conviction for homicide, assault, robbery, arson, any sexual offence (rape, sexual coercion, child molestation, indecent exposure, or sexual harassment), illegal threats, or harassment.

† Inpatient or outpatient International Classification of Diseases diagnosis in patient register.

‡ Dispensed within the last 6 months.

§ Drugs used in addictive disorders.

¶ Welfare or disability benefits.

**Figure 2:**
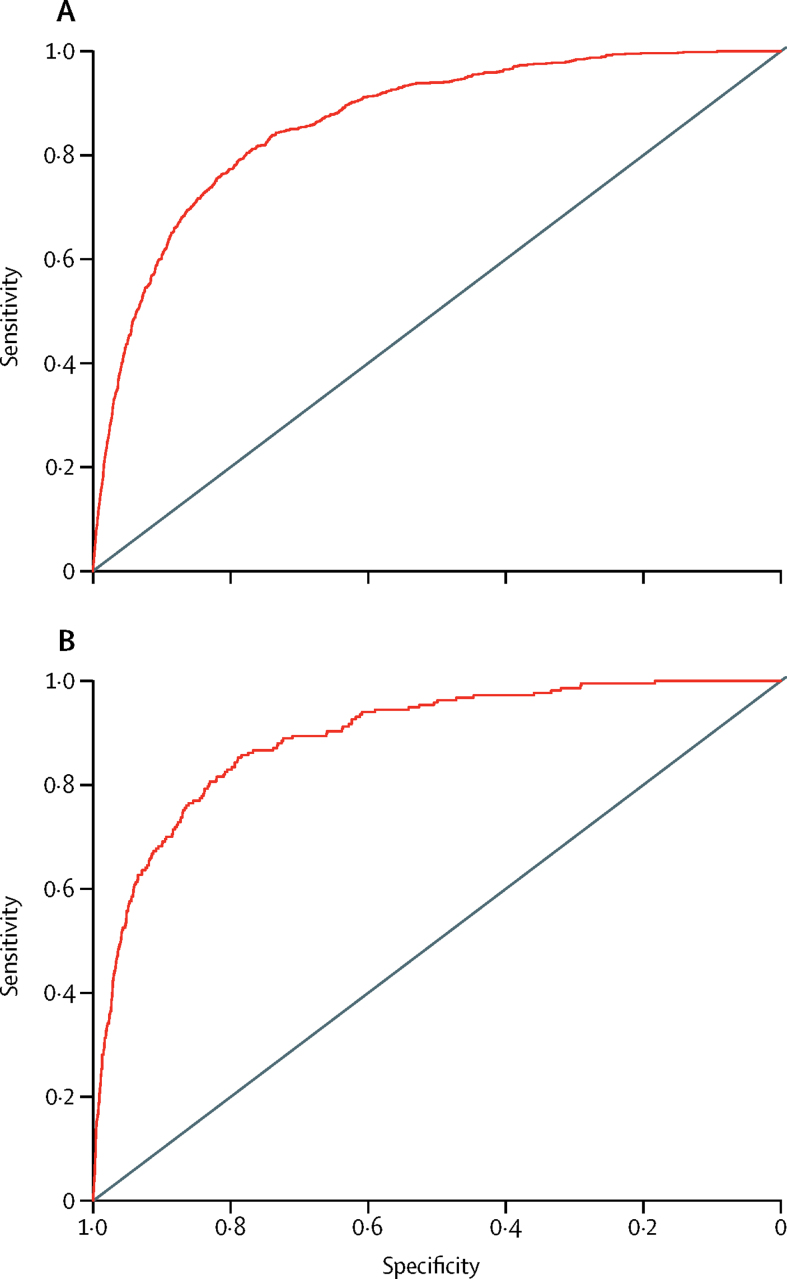
Model discrimination in the (A) derivation and (B) external validation samples

**Figure 3:**
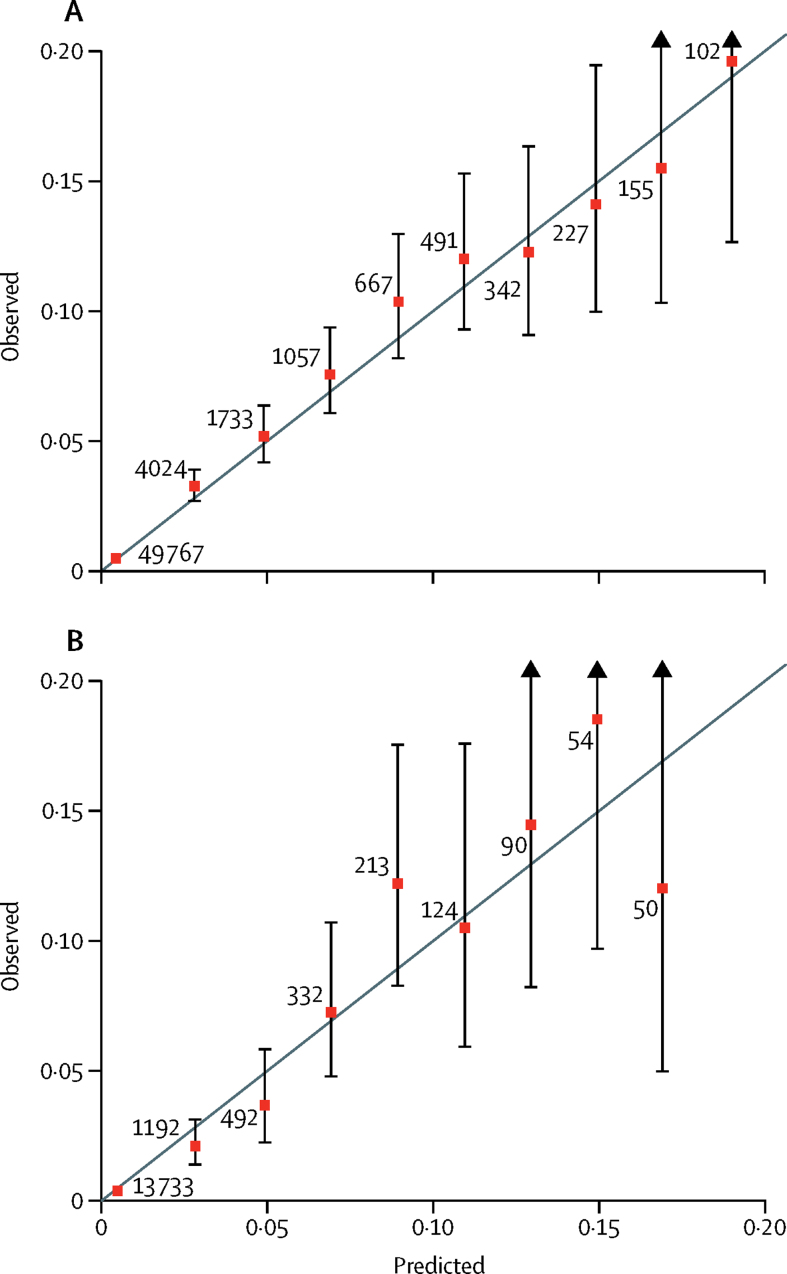
Predicted and observed risks of violent crime in the (A) derivation and (B) external validation samples Individuals are grouped by predicted probability, numbers are the number of individuals in each grouping, and error bars are 95% CIs for the proportion of events in each group.

The model showed good overall discrimination on the basis of the results from both internal validation with use of bootstrapping (c-index 0·86 [95% CI 0·84–0·89]; Brier score 0·0132; net reclassification index 1·14) and external validation (c-index 0·89 [0·85–0·93]; Brier score 0·0120; net reclassification index 1·28). When we used the prespecified 5% risk cutoff for violent crime in 1 year, the sensitivity was 49% (95% CI 45–52) and the specificity was 94% (94–94) in internal validation. The sensitivity in external validation was slightly higher (62% [55–68]) than in internal validation, with the same specificity (94% [93–94]). The positive predictive value was 11% and the negative predictive value was more than 99%. The 2 × 2 tables used to derive sensitivity are shown in the appendix (p 10). Receiver operating characteristic curves are shown in figure 2. Calibration plots indicate adequate calibration of the predicted probabilities against observed proportions of violent offending (figure 3).

We applied the coefficients to develop a web calculator called the Oxford Mental Illness and Violence tool [OxMIV]), which is free to use. This tool provides both a risk classification (low or high) and a probability of violent offending within the next 12 months. A beta version of the online risk calculator for violent offending (based on the coefficients in the appendix [p 8]) can be found online. If missing values are present, this calculator reports the upper and lower range of estimates of risk.

## Discussion

We have described the development of a clinical prediction score and web calculator (OxMIV) for risk of committing violent crime in individuals with diagnoses of schizophrenia spectrum or bipolar disorder with good measures of discrimination and calibration. We developed a 16-item model from prespecified criminal history, sociodemographic, and clinical risk factors. The prediction score is brief and simple to use, relies on information that can be routinely collected, and is able to stratify individuals into high-risk and low-risk groups. It is primarily intended as an adjunct to clinical decision making and to anchor decisions on violence risk using an evidence-based approach, which will need to be complemented by individualised and contextual factors.^22^

Current risk assessment tools predicting violence risk in psychiatric populations are limited by being time-consuming, requiring training, having direct costs in most cases, and randomised controlled trial evidence^23^ that instruments based on structured clinical judgment do not improve patient outcomes. Despite these limitations, they are widely used in general and forensic psychiatry.^24^ To our knowledge, none of the current approaches use web-based calculators, are free to use, and incorporate treatment information. By contrast, OxMIV addresses these limitations, and as the derivation sample was based on 58 771 patients, it provides substantially improved precision for identified risk factors. The performance statistics of OxMIV are better than are those reported for other instruments currently used in mental health. For violent crime over 1 year, the model had a sensitivity of 62% and a specificity of 94% in the external validation sample with use of prespecified risk thresholds. The positive predictive value was 11% and the negative predictive value was more than 99%.

Authors of a 2011 systematic review^9^ found that only two instruments had been validated in 861 patients with psychosis, with areas under the curves (AUCs) that ranged from 0·60 to 0·77, with little other performance information. By contrast, the overall c-index (equivalent to an AUC) of OxMIV was 0·89 in the external validation. A broad review^6^ of all violence instruments used in criminal justice reported a median AUC of 0·72 (IQR 0·68–0·78), with a sensitivity higher than that of OxMIV, at 92%, but a specificity lower, at 36%, with these differences in sensitivity and specificity being explained by different thresholds used in criminal justice populations and in the population of this study. Authors of a review^25^ of commonly used cardiovascular risk scores reported AUCs in the range of 0·70 to 0·75. Another limitation of current approaches is that they might increase stigma, particularly by overestimating risks. More precision than at present about future probabilities of violent crime could partly address this limitation, as well as more awareness of the epidemiological evidence for actual violence over a 12 month period (figure 1).

**Figure 4:**
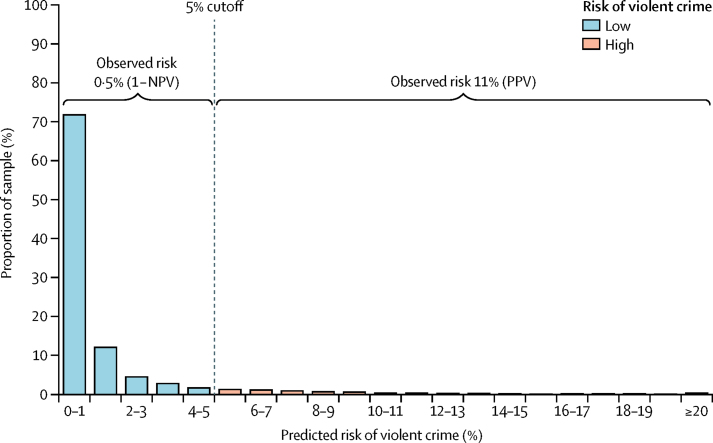
Observed and predicted risk of violent crime in severe mental illness NPV=negative predictive value. PPV=positive predictive value.

One clinical implication of OxMIV is that it could be used to screen for low violence risk in general adult psychiatric services. This use is facilitated by the high negative predictive value (which was was in fact 99·5%)—in other words, of those identified as low risk, 199 of 200 did not in fact offend violently within 1 year. But the low positive predictive value of the tool means that it should not be used to predict violent crime as only around one in 10 of those identified as high risk will actually violently offend. Rather, it can identify patients at higher than average risk (figure 4). However, as OxMIV's sensitivity was 62% using the 5% threshold, it can be used to stratify patients into low-risk and high-risk groups because nearly two-thirds of all those who do violently offend will be picked up by this tool. If the consequences of identification of someone at high risk are not harmful, then this identification has the potential to reduce violence risk. At the same time, this identification cannot be used to detain individuals or extend their detention in the absence of other clinical factors and detailed assessment.

Use of a 5% threshold was based on 1 year postdiagnosis incidence data,^1^ although violence incidence in this sample was lower than that in the previous data as it included individuals at random points after diagnosis, meaning that individuals were older than in previous studies. Nevertheless, the 5% threshold might be considered low by some clinicians, but thresholds higher than 5% will be subject to more false negatives than those of 5% or lower. Broadly, use of this score provides a framework to make decisions between patients, carers, and health-care staff, and allow for anchoring of clinical risk in evidence, particularly as clinicians might overestimate risk.^5^ Furthermore, it could replace tools that are developed by local or regional general adult psychiatric services that have no supporting evidence and are often mandatory for clinicians to complete.

In terms of administration of this prediction score, including its communication to individuals and linkage to treatment, it is important to emphasise that it is only an adjunct to clinical decision making and that clinical judgment should supplement it with relevant individual factors. One strength is that any health-care professional can use OxMIV, including nursing, psychology, and medical staff, in primary and secondary care, but it should not be administered by non-clinical staff as it relies on diagnostic and treatment information and should only be part of a wider clinical assessment that considers other individual factors. The tool can be used at any point in a patient's pathway, apart from in forensic psychiatric patients and released prisoners,^26^ as baseline risks and the effects of risk factors will be different. Some of the items might not be routinely available, and we have provided for the possibility of scoring unknown for the socioeconomic and parental items. However, the risk factors included underscore the importance of taking a full psychiatric history to assist in prognosis.

Strengths of our study include that it was based on a total cohort of all patients with diagnosed schizophrenia spectrum or bipolar disorder, with high-quality registers being linked to provide information about covariates and outcomes. Unlike previous risk assessment instruments,^27^ we have reported measures of discrimination and calibration in both derivation and external validation populations. Predictive accuracy was similar between the derivation and external validation samples, with the external validation sample being geographically separated from the derivation one. This finding would suggest that the model has the potential to be applied to different populations. Additionally, our shrinkage estimate was 99%, which is higher than the 85% that is recommended.^16^ Another methodological strength was use of imputation to replace missing data, which is new in the field of violence risk assessment. Finally, we have provided a free web calculator version for clinical staff.

A limitation of the study is validation in one country. Prevalence estimates for diagnoses of psychotic disorders vary minimally across European countries and the USA.^28^, ^29^, ^30^ In relation to the outcome of violent offending, police-recorded rates of serious violent offences, such as assault, robbery, and rape, have been shown to be fairly similar across high-income countries.^31^ Some miscalibration occurred for individuals at the highest risk at 1 year, but only a small number of individuals had a very high predicted risk, and predicted scores were not consistently higher or lower than observed ones. Nevertheless, we dealt with this miscalibration in the calculator by including a maximum risk of 20% so that any individuals with a predicted risk of 20% or higher all receive the same risk estimate (≥20%). We did not include migration data, which could lead to reduced time at risk for some individuals. However, the effects are likely to be minimal—in a related cohort of patients with schizophrenia spectrum disorders, 0·8% emigrated within 12 months of diagnosis.^1^ Being on medication for alcohol and drug misuse was associated with increasing risk as it was probably confounded by indication and acted as a proxy for severe comorbidity. Furthermore, our model does not include information about risk factors that could be collected in an interview, including specific symptoms,^32^ premorbid conduct problems, anger, victimisation, and comorbid personality disorder, which might further enhance the performance of the tool, but at the cost of making it more complex and time-consuming than without inclusion of this information. We did not consider comorbid personality disorders as their validity in Swedish registers is not known, but is likely to be low. As the tool mostly contains static factors, it should not be used to monitor within-individual changes in risk, but should be used as a cross-sectional score at a particular point in time. Some specific items, such as personal income and benefit receipt, might not be easily generalisable, but we have allowed for them to be scored as unknown and in the fitted model they were fairly weak predictors of violent offending. The possibility to include missing data addresses one previously highlighted concern with existing structured instruments.^5^

An important issue is the implication of being labelled high risk and potential misuses, which could include restrictions on freedom (such as detention in hospital) and further stigma. The tool always needs to be used in conjunction with clinical decision making, and the ethics of deprivation of liberty versus risk to others should be carefully considered.^33^ Although the tool is freely available online, which allows for its widespread use in clinical services, this easy accessibility risks that it could be used for the wrong purposes and in the wrong contexts. Balancing of these issues will remain a challenge, and clear guidelines on the tool's intended population and how it should be used need to be established and regularly updated. Another limitation is use of violent crime as the primary outcome, which, although generalisable (as definitions are common across countries), with clear effects on public health, the absolute rates reported for violent crime are lower than for any violence, and the risk calculator provides a conservative estimate of violence risk. Additionally, the tool should not be used to assess risk of violence before hospital discharge in inpatients with psychiatric disorders, and separate models for subgroups of violent crime were not feasible. Future research should assess whether or not use of this prediction model improves outcomes for individuals with severe mental illness by reducing their risk of violent offending.
