# Supplementary material for: Identification of low risk of violent crime in severe mental illness with a clinical prediction tool (Oxford Mental Illness and Violence tool [OxMIV]): a derivation and validation study
Source: Lancet Psychiatry. 2017 Jun;4(6):461–8. doi: 10.1016/S2215-0366(17)30109-8 (PMC5447135; doi:10.1016/S2215-0366(17)30109-8)
Supplement: Supplementary appendix [file mmc1.pdf]

THE LANCET
Psychiatry

Supplementary appendix

This appendix formed part of the original submission and has been peer reviewed. We post it as supplied by the authors.

Supplement to: Fazel S, Wolf A, Larsson H, Lichtenstein P, Mallett S, Fanshawe TR. Identification of low risk of violent crime in severe mental illness with a clinical prediction tool (Oxford Mental Illness and Violence tool [OxMIV]): a derivation and validation study. *Lancet Psychiatry* 2017; published online May 4. http://dx.doi.org/10.1016/S2215-0366(17)30109-8.

Appendix

Risk factor definitions

From the National Crime Register, we obtained information on any previous violent crime conviction (binary). From the Longitudinal Integration Database for Health Insurance and Social Studies, we obtained socio-economic factors at the year of patient episode. Civil status (ever married versus never married) and benefit receipt (comprising welfare and/or disability benefits) were classified as binary variables. Number of years in education was recoded as a categorical variable, and personal disposable income split into deciles of the distribution in the entire Swedish population. Neighbourhood deprivation was measured as a standardized score of the overall degree of socio-economic deprivation in an individual's residential area, and also split into deciles of the population distribution. These variables were included as deciles rather than as continuous variables so that the same final model could be used in settings in which alternative measures of income or deprivation are used.

We identified diagnoses of psychiatric disorders from the National Patient Register, which provides diagnoses for all inpatient psychiatric hospital admissions in Sweden since 1973 and outpatient care since 2001, according to the International Classification of Diseases (Eighth Revision [ICD-8], 1973-1986; Ninth Revision [ICD-9], 1987-1996; or Tenth Revision [ICD-10], 1997-2009). We investigated the following specific/groups of psychiatric disorders: (1) diagnostic category of severe mental illness in the current patient episode - as schizophrenia-spectrum disorders (ICD-8: 295, 297-299; ICD-9: 295, 297-299 excl. 299A; ICD-10: F20-F29) and bipolar disorders (296 excl. 296.2; 296 excl. 296D; F30-F31), (2) whether this was an inpatient or outpatient episode (3) any previous alcohol use disorder (ICD-8: 291, 303; ICD-9: 291, 303, 305A; ICD-10: F10); (4) any previous drug use disorder (304; 292, 304, 305 excl. 305A; F11-F19), (5) any previous comorbid depression in schizophrenia-spectrum disorder patients (296.2, 300.4; 296D, 300E, 311; F32-F34.1), (6) length of first inpatient stay (>7 days, or ≤ 7 days), (7) number of previous patient episodes for bipolar or schizophrenia (>7 episodes, or ≤ 7), and (8) previous episodes of self-harm based on patient registers (980-989; 980-989; Y10-Y34). Diagnoses of psychiatric disorders were all recorded as binary variables and (apart from current episode diagnosis) based on any lifetime diagnosis prior the current episode.

We obtained data on dispensed medication from the Swedish Prescribed Drug Register for all cases. Recent treatment was defined as dispensed medication within the 6-month period immediately before the index date of diagnosis. Four binary variables for recent treatment were used: (1) antipsychotics (ATC codes N05A [excluding lithium]), (2) mood stabilizers (sodium valproate

N03AG01, lamotrigine N03AX09, carbamazepine N03AF01, oxcarbazepine N03AF02, lithium N05AN01), (3) antidepressants (N06A codes), and (4) drugs used in addictive disorders (N07BB and N07BC codes).

We further identified family members (parents and siblings) of patients through the Multi-Generation Register to extract the following binary historical variables (i.e. before the current episode): (1) parental drug or alcohol use, (2) parental violent crime, (3) sibling violent crime, (4) parental psychiatric hospitalization, (5) parental suicide, and (6) recent death of a family member (within six months preceding discharge). Finally, we included a binary variable on whether the patient shares their household with any children.

Continuous variables were included in the model as linear terms as there was not strong evidence of departure from linearity between continuous variables and the log-odds of the outcome. Variables split into deciles were included as categorical variables. Interactions between risk factors were not considered. Length of first inpatient stay and number of previous episodes were dichotomised in a pre-specified way for ease of interpretation. Measures of income and deprivation were transformed into deciles for generalisability to other populations.

Statistical methods

Statistical analysis was based on logistic regression, adjusting for risk factors as described below. The effects of death and emigration within the follow-up period were ignored as the objective was to predict violent offending within one year irrespective of whether these events occurred, and based only on information available at the time of episode. The statistical analysis plan specified that the model would allow for clustering of individuals within the same family. Clustering was negligible as there were few individuals from the same family in the dataset, and so the clustering effect was removed from the model.

Adjustment for risk factors

Based on existing evidence into criminal history, socio-demographic and clinical factors,[1,2] we grouped variables *a priori* on the anticipated strength of association with the outcome in decreasing levels of priority (Table 1).[3,4] All variables were categorised in this way in a protocol before any statistical analysis was carried out (see appendix p 3 for description of variable groups).

Missing data

Covariates with more than 30% missing data were excluded (e.g. Body Mass Index, other physical characteristics variables, and IQ). Further, IQ information was missing for women and around half of men with schizophrenia-spectrum disorders as it is routinely collected on conscription. An exception was made for the recent treatment variables, which were unavailable before 2006 only because the Prescribed Drug Register was not available: the missingness mechanism was thus known and thought to be unrelated to the missing values themselves. Missing data was imputed via multiple imputation using chained equations (with twenty imputations) using a regression model that used as explanatory variables all other risk factors that were candidates for inclusion in the model, and the outcome variable.[5] Estimates of coefficients in the final prediction rule were obtained by pooling across imputations, using standard methodology.[6]

Validation and goodness of fit

The internal validity of the model was assessed using bootstrapping to assess its predictive accuracy.[7] Bootstrapping was used to create 100 samples drawn with replacement from the derivation dataset; more bootstrap samples were not required as model performance measures were found to be very similar in different samples. A heuristic shrinkage estimate (model χ^2 – degrees of freedom)/ model χ^2) was calculated to assess the generalisability of the model.[8] Model performance was also assessed

using the external validation sample. The concordance index, Brier score, net reclassification index, and sensitivity and specificity were calculated using the predicted probabilities obtained by averaging the predictions from each of the multiply-imputed datasets, each applied to the final model.

Risk factor groups

Group 1 consists of variables thought necessary to include in the statistical model regardless of statistical significance, in order to ensure face validity and to reduce the number of candidate predictors used in the variable selection procedure described below. For the majority of these risk factors, there was evidence from previous research of an association with the outcome measure.

Group 2 consists of variables thought likely to show an association with outcomes, but which are not required to be included to achieve face validity. These variables were included in a backwards stepwise selection procedure, with Group 1 variables always retained in the model, such that they were sequentially rejected in order of p-value until no Group 2 variables remained with p-values greater than 0.1.

Group 3 variables, for which there was weaker prior evidence of an association with the outcome, were subsequently included in a similar stepwise variable selection procedure, retaining all Group 1 and Group 2 variables that had already been included.

Appendix Table 1 – Geographical regions

Regions are primarily based on the counties of Sweden, derived from the first two digits of the SAMS code. Exceptions are the municipalities of Gothenburg and Malmö, which are separated from their respective counties, and Stockholm municipality, which is separated from its county and sub-divided into northern and southern parts by identifying each SAMS area with the historical province in which it is located. Regions were allocated to four groups, which are proxy measures of urban/rural status: the four urban areas (Group 1); the three counties in which the urban areas are located (Group 2); four counties with low population (Group 3); and all other counties (Group 4). The external validation sample was selected by randomly, with equal probability, choosing one region from each of the first three groups, and selecting sequentially from the fourth group until a minimum of 180 violent crime cases in total had been reached.

**Group 1**	**Group 2**	**Group 3**	**Group 4**
Major urban centres	Counties with major urban centres removed	Counties with small population	Counties with medium population
Stockholm City North	Stockholm County Other	Kronoberg	Uppsala
Stockholm City South		Gotland	Södermanland
Malmö	Skåne Other	Blekinge	Östergötland
Gothenburg	Västra Götaland Other	Jämtland	Jönköping
			Kalmar
			Halland
			Värmland
			Örebro
			Västmanland
			Dalarna
			Gävleborg
			Västernorrland
			Västerbotten
			Norrbotten

Appendix Table 2. Tripod Checklist[9]

Section/Topic			Checklist Item	Page
**Title and abstract**				
Title	1	D;V	Identify the study as developing and/or validating a multivariable prediction model, the target population, and the outcome to be predicted.	1
Abstract	2	D;V	Provide a summary of objectives, study design, setting, participants, sample size, predictors, outcome, statistical analysis, results, and conclusions.	2
**Introduction**				
Background and objectives	3a	D;V	Explain the medical context (including whether diagnostic or prognostic) and rationale for developing or validating the multivariable prediction model, including references to existing models.	3-4
	3b	D;V	Specify the objectives, including whether the study describes the development or validation of the model or both.	3-4
**Methods**				
Source of data	4a	D;V	Describe the study design or source of data (e.g., randomized trial, cohort, or registry data), separately for the development and validation data sets, if applicable.	5
	4b	D;V	Specify the key study dates, including start of accrual; end of accrual; and, if applicable, end of follow-up.	5
Participants	5a	D;V	Specify key elements of the study setting (e.g., primary care, secondary care, general population) including number and location of centres.	5
	5b	D;V	Describe eligibility criteria for participants.	5
	5c	D;V	Give details of treatments received, if relevant.	6
Outcome	6a	D;V	Clearly define the outcome that is predicted by the prediction model, including how and when assessed.	6
	6b	D;V	Report any actions to blind assessment of the outcome to be predicted.	-
Predictors	7a	D;V	Clearly define all predictors used in developing or validating the multivariable prediction model, including how and when they were measured.	5-6, 18
	7b	D;V	Report any actions to blind assessment of predictors for the outcome and other predictors.	-
Sample size	8	D;V	Explain how the study size was arrived at.	5
Missing data	9	D;V	Describe how missing data were handled (e.g., complete-case analysis, single imputation, multiple imputation) with details of any imputation method.	7-8
Statistical analysis methods	10a	D	Describe how predictors were handled in the analyses.	5-6
	10b	D	Specify type of model, all model-building procedures (including any predictor selection), and method for internal validation.	6-7
	10c	V	For validation, describe how the predictions were calculated.	8
	10d	D;V	Specify all measures used to assess model performance and, if relevant, to compare multiple models.	8
	10e	V	Describe any model updating (e.g., recalibration) arising from the validation, if done.	-
Risk groups	11	D;V	Provide details on how risk groups were created, if done.	8-9
Development vs. validation	12	V	For validation, identify any differences from the development data in setting, eligibility criteria, outcome, and predictors.	8
**Results**				
Participants	13a	D;V	Describe the flow of participants through the study, including the number of participants with and without the outcome and, if applicable, a summary of the follow-up time. A diagram may be helpful.	10
	13b	D;V	Describe the characteristics of the participants (basic demographics, clinical features, available predictors), including the number of participants with missing data for predictors and outcome.	10, 18
	13c	V	For validation, show a comparison with the development data of the distribution of important variables (demographics, predictors and outcome).	24
Model development	14a	D	Specify the number of participants and outcome events in each analysis.	10
	14b	D	If done, report the unadjusted association between each candidate predictor and outcome.	-
Model specification	15a	D	Present the full prediction model to allow predictions for individuals (i.e., all regression coefficients, and model intercept or baseline survival at a given time point).	23
	15b	D	Explain how to the use the prediction model.	10
Model performance	16	D;V	Report performance measures (with CIs) for the prediction model.	10
Model-updating	17	V	If done, report the results from any model updating (i.e., model specification, model performance).	-
**Discussion**				
Limitations	18	D;V	Discuss any limitations of the study (such as nonrepresentative sample, few events per predictor, missing data).	12-13
Interpretation	19a	V	For validation, discuss the results with reference to performance in the development data, and any other validation data.	11-12
	19b	D;V	Give an overall interpretation of the results, considering objectives, limitations, results from similar studies, and other relevant evidence.	11-12
Implications	20	D;V	Discuss the potential clinical use of the model and implications for future research.	11-13
**Other information**				
Supplementary information	21	D;V	Provide information about the availability of supplementary resources, such as study protocol, Web calculator, and data sets.	10
Funding	22	D;V	Give the source of funding and the role of the funders for the present study.	14

*Items relevant only to the development of a prediction model are denoted by D, items relating solely to a validation of a prediction model are denoted by V, and items relating to both are denoted D;V. We recommend using the TRIPOD Checklist in conjunction with the TRIPOD Explanation and Elaboration document.

Appendix Table 3 – untransformed model coefficients

**Variable**	**Coefficient**
constant	-3.7273
Sex – male	0.8400
Age (per 10 years)	-0.0468
Previous violent crime	1.6149
Previous drug use	0.3737
Previous alcohol use	0.5603
Previous self-harm	0.2049
Educational level	
Upper secondary	-0.1264
Post-secondary	-0.0703
Parental drug or alcohol use	0.1044
Parental violent crime	0.1464
Sibling violent crime	-0.1076
Recent treatment – antipsychotic	-0.4708
Recent treatment – antidepressant	-0.2209
Recent treatment – dependence	0.5783
Inpatient at time of episode	0.3143
Benefit receipt	0.3508
Personal income	
2nd decile	-0.1829
3rd decile	-0.1708
4th decile	-0.3646
5th decile	-0.1688
6th decile	-0.0825
7th decile	-0.4445
8th decile	0.2834
9th decile	-0.2237
10th decile	-0.1233

To calculate predicted probability for an individual:

1) Calculate X = -3.7273 + 0.8400*Male – 0.0468*Age + 1.6149*Previous violent crime + …

2) Calculate predicted probability = 1 / [1 + exp(-X)]

3) If predicted probability > 5%, individual is classified at high risk; otherwise individual is classified at low risk

Appendix Figure 1 - Scatterplot of probability of violent crime against age (individuals grouped into five-year age bands), with loess fitted curve

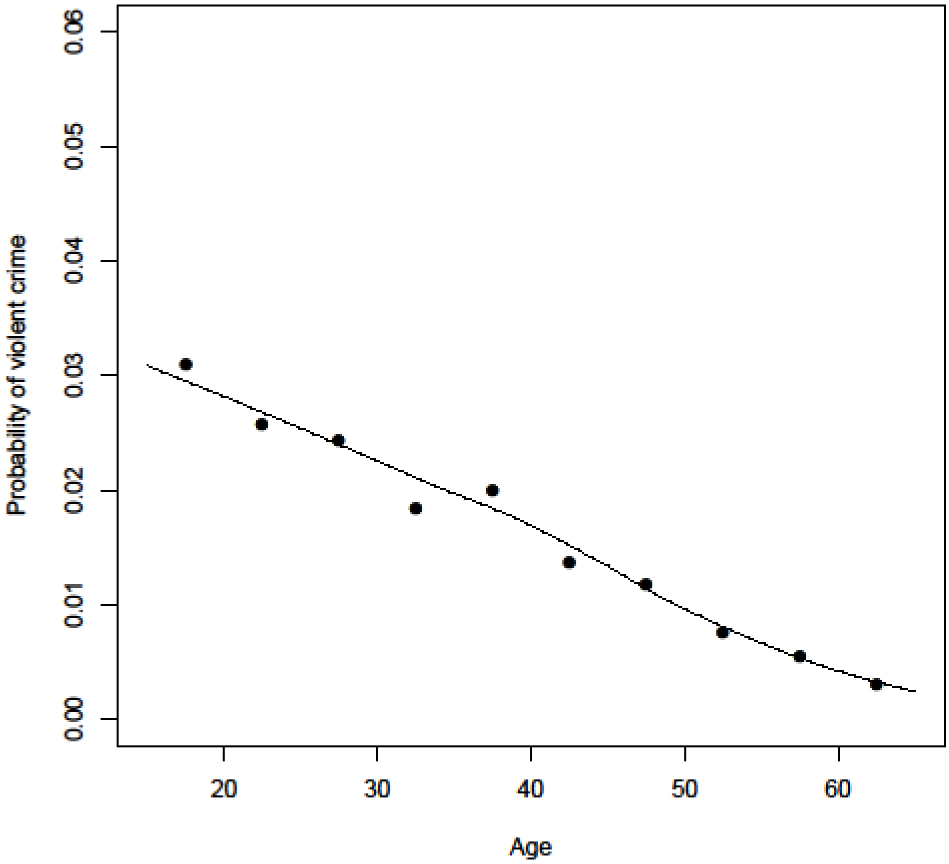

Appendix Table 4: Two by two tables used to derive estimate of sensitivity and specificity in derivation sample (after multiple imputation) and validation sample

a) Derivation sample

		Outcome		
		+	−	Total
Prediction	+	405	3572	3977
	−	425	54369	54794
	Total	830	57941	58771

b) Validation sample

		Outcome		
		+	−	Total
Prediction	+	134	1050	1184
	−	83	15120	15203
	Total	217	16170	16387

Note that the 2x2 table for the derivation sample may not correspond exactly to the summary statistics obtained for internal validation, which was based on an average over bootstrapped data samples.

Appendix references

1. Witt K, Van Dorn R, Fazel S. Risk Factors for Violence in Psychosis: Systematic Review and Meta-Regression Analysis of 110 Studies. *PloS one* 2013; 8(2):e55942.
2. Bonta J, Blais J, Wilson HA. A theoretically informed meta-analysis of the risk for general and violent recidivism for mentally disordered offenders. *Aggression and Violent Behavior* 2014; 19(3):278-87.
3. Royston P, Moons KG, Altman DG, Vergouwe Y. Prognosis and prognostic research: developing a prognostic model. *Bmj* 2009; 338(b604.
4. Royston P, Sauerbrei W. Multivariable model-building. *Chichester: Wiley* 2008.
5. Sterne J, White I, Carlin J, et al. Multiple imputation for missing data in epidemiological and clinical research: potential and pitfalls. *Bmj* 2009; 338(157-60.
6. Barnard J, Rubin D. Small-sample degrees of freedom with multiple imputation. *Biometrika* 1999; 86(948-55.
7. Harrell FE, Lee KL, Mark DB. Tutorial in biostatistics multivariable prognostic models: issues in developing models, evaluating assumptions and adequacy, and measuring and reducing errors. *Statistics in Medicine* 1996; 15(361-87.
8. Van Houwelingen J, Le Cessie S. Predictive value of statistical models. *Statistics in Medicine* 1990; 9(11):1303-25.
9. Collins GS, Reitsma JB, Altman DG, Moons KG. Transparent reporting of a multivariable prediction model for individual prognosis or diagnosis (TRIPOD): the TRIPOD statement. *Bmj* 2015; 350(g7594.
